# Predictors of support for anti-weight discrimination policies among Canadian adults

**DOI:** 10.3389/fpubh.2023.1060794

**Published:** 2023-04-17

**Authors:** Matthew Levy, Vida Forouhar, Iyoma Y. Edache, Angela S. Alberga

**Affiliations:** ^1^Department of Health, Kinesiology and Applied Physiology, Concordia University, Montreal, QC, Canada; ^2^School of Population and Public Health, University of British Columbia, Vancouver, BC, Canada

**Keywords:** weight discrimination policy, weight bias, weight stigma, discrimination, obesity, public policy

## Abstract

**Introduction:**

Weight discrimination of individuals with overweight or obesity is associated with adverse mental and physical health. Weight discrimination is prevalent in many sectors such as within workplaces, where individuals with overweight and obesity are denied the same opportunities as individuals with lower weight status, regardless of performance or experience. The purpose of this study was to understand the Canadian public's support or opposition of anti-weight discrimination policies and predictors of support. It was hypothesized that Canadians will show support of anti-weight discrimination policies to some extent.

**Methods:**

A secondary analysis was conducted on a previous cross-sectional sample of Canadian adults (*N* = 923, 50.76% women, 74.4% White) who responded to an online survey assessing weight bias and support of twelve anti-weight discrimination policies related to societal policies (e.g., implementing laws preventing weight discrimination) and employment-related policies (e.g., making it illegal to not hire someone due to their weight). Participants completed the Causes of Obesity Questionnaire (COB), the Anti-Fat Attitudes Questionnaire (AFA) and the Modified Weight Bias Internalization Scale (WBIS-M). Multiple logistic regressions were used to determine predictors of policy support.

**Results:**

Support for policies ranged from 31.3% to 76.9%, with employment anti-discrimination policies obtaining greater support than societal policies. Identifying as White and a woman, being over the age of 45 and having a higher BMI were associated with an increased likelihood of supporting anti-weight discrimination policies. There were no differences between the level of support associated with attributing obesity to behavioral or non-behavioral causes. Explicit weight bias was associated with a reduced likelihood of supporting 8/12 policies. Weight Bias Internalization was associated with an increased likelihood of supporting all societal policies but none of the employment policies.

**Conclusions:**

Support for anti-weight discrimination policies exists among Canadian adults, and explicit weight bias is associated with a lower likelihood of supporting these policies. These results highlight the need for education on the prevalence and perils of weight discrimination which may urge policy makers to consider weight bias as a form of discrimination that must be addressed. More research on potential implementation of anti-weight discrimination policies in Canada is warranted.

## 1. Introduction

Weight discrimination, defined as the unfair treatment of individuals due to their weight, is pervasive throughout all facets of society (1), including education, healthcare, employment, and in interpersonal relationships and public settings (2). For example, within the workplace, individuals with overweight or obesity are often not given the same opportunities, compensations or promotions as individuals with lower weight status, regardless of performance, education or experience (1, 3–5). Among U.S. women classified as having below average weight, gaining 25 pounds predicted a decreased annual salary exceeding $13,000 (6). Contrarily, for men in similar weight categories, the same weight gain predicted an increased annual salary of more than $7,500 (6). Similar discriminatory behavior has been documented within the health care system. According to a scoping review, several studies have documented weight-based discrimination and negative attitudes toward patients with overweight or obesity among health care professionals (7). Examples include disrespectful treatment and poor or insensitive communication with patients with overweight or obesity compared to patients classified as having a normal BMI and poor or insensitive communication (7). Health care professionals also make inaccurate assumptions about their patients' health behaviors simply by making judgements about their weight status (7).

Weight discrimination remains one of the leading forms of everyday discrimination experienced by Canadians (8, 9). This is concerning as weight discrimination is associated with a variety of negative mental and physical health measures, including depressive symptoms, anxiety (10–12), disordered eating behaviors (13), future weight gain (14), increased inflammatory markers (15), high-risk health behaviors (16), and physical inactivity (17). Unlike other forms of discrimination (race, gender, age, sexual orientation, physical disability, etc.), which are protected against in Canada's Human Rights Act (18), there are currently no Canadian laws or policies aimed to protect against weight discrimination (19). This prompted the evolution of a public movement aimed at protecting individuals with higher weights from discrimination by classifying obesity as a disability (20). However, there are several controversies that exist surrounding the classification of obesity as a disability. Under the Americans with Disabilities Act, individuals who attempt to take legal action against weight discrimination must prove that their weight is a disability (21). Additionally, classifying obesity as a disability may inadvertently perpetuate weight bias and reinforce discrimination (22). There is evidence of moderate success in classifying “severe obesity” as a disability and seeking protection for those with “severe obesity” (21). However, as individuals with overweight and moderate obesity still remain unprotected, they have sought to pursue legal action against employers for unlawful termination due to their weight status (21). In both Canada and the U.S., discrimination toward individuals with overweight or moderate obesity is legally permitted. The state of Michigan in the U.S. is an exception to this due to legislation prohibiting weight discrimination. There are also only a handful of other U.S. localities that have such policies implemented, such as in San Francisco and Santa Cruz in California (21, 23). Only one study examined the effects of implementing anti-weight discrimination policies on reports of weight discrimination (24). This study assessed the prevalence of weight discrimination in Michigan (after the law to protect against weight discrimination passed) and showed a decrease in reports of weight discrimination by women with obesity (24). Although more research is needed, these few studies showcase the potential of these policies to reduce the prevalence of weight discrimination. Legislation against weight discrimination may help to reduce the frequency of weight-based employment discrimination as well as weight-based bullying or teasing in schools. Although research is needed to examine the psychological health benefits of anti-weight discrimination policy implementation, it has been proposed that they could improve the psychological wellbeing of individuals who self-stigmatize or who experience frequent weight discrimination, by delegitimizing discrimination and discouraging individuals from blaming themselves for their weight status (24, 25). Despite the potential positive psychosocial impact of anti-weight discrimination policies, they have an increased likelihood of being implemented if they have the public's support. Therefore, an evaluation of public support of anti-discrimination policy is warranted to provide potential justification for policy implementation.

Previous studies have assessed public support of anti-weight discrimination policies among adults in the U.S. (21, 23, 26–28), Germany (29), Australia (21, 28), Iceland (21), France (28), and the UK (28). Results from these previous studies demonstrated support for anti-weight discrimination policies and showed that variables such as age, race, gender, BMI, income, political orientation, experiences of weight stigma, WBI, and the beliefs regarding the causes of obesity significantly predicted policy support or opposition (21, 23, 26–29). Particularly, younger age, higher BMIs, identifying as a woman, experiencing WBI and weight stigma, a liberal political orientation, and endorsing physiological causes of obesity predicted support of these policies. One of these previous studies examined public support of anti-weight discrimination polices across four countries, including a sample of Canadian adults (21). However, the Canadian sample of this study was not intended to be representative of the Canadian public as the sample consisted of health professionals who were members of Obesity Canada (formerly known as the Canadian Obesity Network) and known for their advocacy initiatives to reduce weight bias and discrimination (21). A more recent publication examined support for these policies across six countries, similarly with a sample of Canadian adults, however this study was also limited to a sample of treatment seeking adults belonging to the WW community (28). Consequently, there are currently no studies assessing public support and predictors of support for anti-weight discrimination policies among a sample of Canadian adults taken from the general population. Therefore, the aim of this study was to assess public support of anti-weight discrimination policies and determine predictors of support for these policies in a sample of Canadian adults taken from the general population. It is hypothesized that women, individuals with higher BMI and WBI, older individuals, and those who endorse non-behavioral causes of obesity will have greater support of anti-weight discrimination policies. Furthermore, high explicit weight bias and beliefs in behavioral causes of obesity will predict less support for these policies.

## 2. Methods

### 2.1. Samples and procedures

Data for this study were obtained from a cross-sectional near-representative sample gathered in 2018 by a market research company, Survey Sampling International (SSI). Recruitment quotas for this study were based on age, gender, and province of residence, allowing for a near approximation of Canadian census demographics. Canadians over the age of 18 years old were eligible to participate and were informed of the study purpose, protocol, and incentivization *via* email. The 20-min survey was hosted on SurveyMonkey, an online survey platform. All participants provided informed consent and the research project was approved by Concordia University's Research Ethics Board (ethics certification number: 30009752).

### 2.2. Measures

#### 2.2.1. Demographic questionnaire

The demographic questionnaire included items assessing participants' age, gender, race, annual household income, and self-reported height and weight.

#### 2.2.2. Weight status

Body Mass Index (BMI) was calculated by utilizing the self-reported height and weight provided by participants. BMI was classified using guidelines categorizing participants into weight status groups including underweight (BMI < 18.5 kg/m^2^), normal weight (18.5–24.9 kg/m^2^), overweight (BMI 25.0–29.9 kg/m^2^) and having obesity (BMI > 30 kg/m^2^) (30).

#### 2.2.3. Explicit weight bias

Explicit weight bias was assessed with the Anti-Fat Attitudes Questionnaire (AFA) (31). The thirteen-item questionnaire contained three subscales which assessed different dimensions of explicit weight bias: *Dislike, Fear of Fat* and *Willpower*. The *Dislike* subscale (*n* = 7) assessed negative feelings toward individuals living in larger bodies (e.g., I really don't like obese people much). The *Fear of Fat* subscale (*n* = 3) assessed an individual's own concerns about gaining weight or having a larger body (e.g., I feel disgusted with myself when I gain weight). The *Willpower* subscale (*n* = 3) assessed an individual's beliefs regarding the personal controllability of weight (e.g., Obese people tend to be fat pretty much through their own fault). All items were scored on a ten-point Likert scale (zero = very strongly disagree; nine = very strongly agree). Items were summated and then divided by the specific number of items within each subscale, ultimately deriving a mean score between zero and nine for each subscale. Items were coded so that higher scores are indicative of higher levels of explicit weight bias. In this study, internal consistency for each subscale was relatively high with Cronbach alpha values of 0.88, 0.85, and 0.82 for the *Dislike, Fear of Fat*, and *Willpower* subscales, respectively.

#### 2.2.4. Causes of obesity

The belief regarding the personal controllability or potential causes of obesity was assessed with the Causes of Obesity (COB) Questionnaire, a fourteen-item questionnaire which includes a variety of different potential causes that can contribute to overweight and obesity (32). Each item is scored on a five-point Likert scale (one = not at all important; five = extremely important), whereby higher scores represent a belief that a specific factor is more important in contributing to one's weight status. The items within the questionnaire can be classified into two distinct groups: behavioral causes (e.g., restaurant eating) and non-behavioral causes (e.g., genetic factors) (33). The behavioral causes describe causes of obesity that are more within the control of the individual, while the non-behavioral causes of obesity are not within the control of the individual, such as physiological or environmental causes. A mean score for each subscale is derived by summating the total score and dividing it by the number of items within each respective subscale. In this study, internal consistency for the COB Questionnaire was relatively high with a Cronbach alpha value of 0.89. The internal consistency for the behavioral and non-behavioral causes subscales were also relatively high with a Cronbach alpha value of 0.80 and 0.83, respectively.

#### 2.2.5. Weight bias internalization

The Modified Weight Bias Internalization Scale (WBIS-M) was utilized to assess the extent to which an individual applies negative weight stereotypes onto themselves and internalizes negative body weight ideals (34). The WBIS-M is an eleven-item questionnaire where items are scored on a seven-point Likert scale (1 = strongly disagree; 7 = strongly agree), with higher scores indicating elevated WBI. An example of an item included in the WBIS is “My weight is a major way that I judge my value as a person.” The total score is divided by the number of items to obtain a mean Weight Bias Internalization (WBI) between one and seven. Previous literature has demonstrated that the first item of the WBIS-M has poor psychometric properties (35). Therefore, in this current study, we utilized the ten-item version of the WBIS-M. The ten-item WBIS-M has been shown to have high internal consistency (35). In the current study, the ten-item WBIS-M had high internal consistency as well with a Cronbach alpha value of 0.94.

#### 2.2.6. Level of support for anti-weight discrimination policies

Participants were asked to rate their support for twelve policies aimed at prohibiting weight discrimination throughout society, and specifically within the workplace (21). The policies evaluated herein were taken directly from Puhl et al. (21). Puhl et al.'s study used some items that were previously researched by their team (4, 27), and other items that were adapted from previous research on legal and public health perspectives of weight discrimination, and studies on legislation prohibiting weight discrimination (36, 37). These items proposed legal measures, civil right actions, and employment regulations aimed to counter widely accepted forms of weight discrimination (21). Participants rated their support for each proposed policy on a five-point Likert scale (1 = strongly disagree; 5 = strongly agree). Rating an agreement of *Agree* or *Strongly Agree* were both coded as being in favor of the proposed policy statement. Policies 1 through 5 were categorized as broad societal policies such as legal and civil rights actions [e.g., The government should penalize (or fine) those who discriminate against persons because of their weight]. Policies 6 through 10 were policies aimed at prohibiting employment-related weight discrimination or weight discrimination within the workplace (e.g., It should be illegal for an employer to terminate or fire a qualified employee because of his or her body weight). Policies 11 and 12 reflected measures that support weight discrimination within the workplace (e.g., Employers should be allowed to assign different salaries to employees based on their body weight). In accordance with other studies in the literature, interpretation and discussion of results pertaining to policies 11 and 12 have been reverse worded to reflect opposition of workplace weight discrimination. The number of participants in favor of each policy were summated to obtain the total number of proposed policies that received agreement from each participant. All of these policies are listed in **Table 2**.

### 2.3. Data analysis

Statistical analyses were conducted using SPSS version 24. Public support of anti-weight discrimination policies was assessed by examining the percentage of participants who supported each specific policy. Predictors of support for each law were analyzed by conducting a series of multiple logistic regressions that tested for predictors of support based on age, gender, race, household income, BMI, Anti Fat Attitudes (AFA) subscales, Causes of Obesity (COB) subscales, and Weight Bias Internalization (WBI). These logistic regressions were adjusted for age, gender, race, and BMI (except for the analyses involving these specific predictors).”

## 3. Results

### 3.1. Descriptive characteristics

The study's sample characteristics are summarized in Table 1. The total sample consisted of primarily White individuals (74.4%), with 48% women, 50% men, and 1% identifying as other. Mean BMI within the total sample was 27.16 ± 7.23 kg/m^2^, whereby 5.7% of participants were classified as having underweight, 34.2% as normal weight, 33.2% as overweight, and 26.9% as having obesity.

**Table 1:** Sample characteristics.

**Measure**	**Total sample (*N* = 923)**
**Age**, ***n*** **(%)**	
18–24	98 (10.6)
25–44	331 (35.9)
45–65	366 (39.7)
65+	128 (13.9)
**Gender**, ***n*** **(%)**	
Men	445 (48.2)
Women	469 (50.8)
Other	9 (1.0)
**Body Mass Index kg/m** ^2^	**27.16** ±**7.23**
Underweight, *n* (%)	53 (5.7)
Normal weight, *n* (%)	316 (34.2)
Overweight, *n* (%)	306 (33.2)
Obesity, *n* (%)	248 (26.9)
**Race/ethnicity**, ***n*** **(%)**	
White	687 (74.4)
Non-white	236 (25.6)
Asian	96 (10.4)
Black/African/Caribbean	27 (2.9)
South Asian	27 (2.9)
Aboriginal	23 (2.5)
Other	19 (2.1)
Middle Eastern	13 (1.4)
Southeast Asian	11 (1.2)
Biracial/Bi-ethnic	9 (1.0)
Hispanic/Latin American	9 (1.0)
Pacific Islander	2 (0.2)
**Income**, ***n*** **(%)**	
< $25,000	175 (19.0)
$25,000–$49,999	255 (27.6)
$50,000–$74,999	224 (24.3)
$75,000+	269 (29.1)
**Causes of obesity**	
Behavioral causes	3.55 ± 0.74
Non-behavioral causes	3.38 ± 0.78
**Anti-fat attitudes**	
Dislike	2.38 ± 1.90
Fear of fat	4.55 ± 2.68
Willpower	4.58 ± 2.20
**Weight Bias Internalization**	3.42 ± 1.51

Causes of obesity: minimum = 1, maximum = 5.

Anti-fat attitudes: minimum = 0, maximum = 9.

Weight Bias Internalization: minimum = 1, maximum = 7.

### 3.2. Support for anti-weight discrimination policies

Support for anti-weight discrimination policies within the total sample ranged from 31.3% to 76.9% (Table 2). Policy 8 received the highest level of support, which states that it should be illegal for an employer to assign lower wages to a qualified employee because of their body weight. Policy 4 received the lowest level of support overall, which advocates to classify obesity as a disability to protect those from experiencing weight discrimination. The majority of participants (77% and 85.0%, respectively) oppose workplace weight discrimination indicated in policies 11 and 12. Most participants agree that individual companies should not have the right to determine whom to hire based on an employee's personal body weight (policy 11) and that employers should not be allowed to assign different salaries to employees based on their body weight (policy 12).

**Table 2:** Support for anti-weight discrimination policies.

**Proposed anti-weight discrimination policy**	**Agreement/support of proposed policies, *n* (%)**
**Societal policies**	**Total sample (*****N*** = **923)**
1. My country should include body weight in our civil rights law in order to protect people from discrimination based on their body weight.	396 (42.9)
2. The government should have specific laws in place to protect people from weight discrimination.	461 (49.9)
3. The government should penalize (or fine) those who discriminate against persons because of their weight.	475 (51.5)
4. Obesity should be considered a disability so that people will be protected from weight discrimination in the workplace.	289 (31.3)
5. Fat persons should be subject to the same legal protections and benefits offered to people with physical disabilities.	355 (38.5)
**Employment policies**	
6. The government should pass the Weight Discrimination in Employment Act to protect employees from discrimination in the workplace based on their body weight.	465 (50.4)
7. It should be illegal for an employer to refuse to hire a qualified person because of his or her body weight.	602 (65.2)
8. It should be illegal for an employer to assign lower wages to a qualified employee because of his or her body weight.	710 (76.9)
9. It should be illegal for an employer to terminate or fire a qualified employee because of his or her body weight.	689 (73.7)
10. It should be illegal for an employer to deny a promotion or appropriate compensation to a qualified employee because of his or her body weight.	701 (75.9)
11. Individual companies should have the right to determine whom to hire based on an employee's personal body weight.	211 (22.9)
12. Employers should be allowed to assign different salaries to employees based on their body weight.	138 (15.0)

### 3.3. Predictors of support for anti-weight discrimination policies

Refer to Table 2 for a list of all policies evaluated and their corresponding assigned policy number.

### 3.4. Societal policies (1–5)

Predictors of support for anti-weight discrimination policies are described in Supplementary Table 3. Out of all five societal policies, gender only predicted support for policy 1, a policy aimed at including weight within a country's civil rights law, whereby men were less likely to support policy 1 compared to women. Income only predicted support for policy 4, a policy aimed at classifying obesity as a disability, whereby those with incomes exceeding $75,000 were less likely to support policy 4 compared to those who made <$25,000. Having an elevated BMI predicted support for policies 1, 2, 4, and 5. Compared to those between the ages of 14–24 years, being older than 65 years old predicted support for policies 3, 4, and 5.

Attribution of overweight and obesity to non-behavioral causes predicted support for all five societal policies, but attributing causes of obesity to behavioral causes only predicted support for the first four societal policies 1 to 4. *Fear of Fat* was associated with an increased likelihood of supporting policies 1 and 2, *Willpower* was associated with a reduced likelihood of supporting policy 3, and *Dislike* was associated with a reduced likelihood of supporting policy 2, but an increased likelihood of supporting policy 4. WBI predicted support of all five societal anti-weight discrimination policies.

### 3.5. Employment policies (6–12)

Gender, age, and income predicted support for policies 7–10. Men were less likely to support these policies compared to women. Older (45–64 and older than 65 years old) and White participants were more likely to support these policies compared to younger (18–24 years old), and non-White participants, respectively. Income predicted support of policies 8 and 9, whereby those who earned more than $50,000 were more likely to support these two policies compared to those who earned <$25,000. Having an elevated BMI predicted support for policies 7, 8 and 9.

Attributing obesity to both behavioral and non-behavioral causes predicted support of policies 6–10. *Dislike* was associated with a reduced likelihood of supporting policies 6–10, *Fear of Fat* was associated with an increased likelihood of supporting policy 8 and *Willpower* was associated with a reduced likelihood of supporting policy 6. Gender, race, *Dislike*, and WBI predicted support for policies 11 and 12. Women were more likely to oppose workplace weight-discrimination compared to men, whereas White participants were more likely to oppose workplace weight-discrimination compared to non-White participants. Having an elevated BMI was associated with an increased likelihood of opposing policy 11.

## 4. Discussion

The results of this study indicate that Canadian adults support anti-weight discrimination policies, with support ranging from 31.3% to 76.9%. Identifying as a woman, White, being over the age of 45, and having an elevated BMI were all associated with an increased likelihood of supporting several societal and employment-related anti-weight discrimination policies. Attributing the causes of obesity to behavioral and non-behavioral causes predicted support for 9 out of 12 policies. However, considering that both behavioral and non-behavioral subgroups of the COB questionnaire predicted support, we cannot determine the role of weight controllability beliefs in predicting support for anti-weight discrimination policies, which deserves future research attention. Supporting policy 5, which states that individuals with overweight and obesity should have the same legal protection as those with physical disabilities, was only predicted by non-behavioral causes. Supporting the notion that employers should not have the right to assign different salaries or hire employees based on body weight (policies 11 and 12), was not predicted by the beliefs about the causes of obesity. In terms of explicit weight bias, *Dislike* was significantly associated with a reduced likelihood of supporting six policies aimed at reducing weight discrimination and significantly predicted the support of policies 11 and 12. WBI significantly predicted the support of all five societal policies. In terms of the employment policies, WBI only significantly predicted the opposition of policies 11 and 12 aimed at upholding the legality of weight discrimination. In terms of the demographic predictors, gender predicted policy support, whereby women were more likely to support policies compared to men. Participants with overweight and obesity were also more likely to support anti-weight discrimination policies than those classified as having normal weight. Previous studies have shown that women and people living with overweight or obesity experience more weight bias, stigma, and discrimination than men and people without overweight or obesity respectively (38). These trends are likely due to society's idealization surrounding beauty and thinness and the public's perceived stereotypes toward those with overweight and obesity (38). Therefore, those who experience increased levels of weight discrimination are more likely to support policies aimed to reduce weight discrimination.

Only one known multinational study has examined support of anti-weight discrimination policies among a Canadian convenience sample of adults, which also assessed support of these policies among samples from Iceland, Australia, and the U.S. (21). Results from this previous study demonstrated that Canadian adults (*N* = 621) expressed greater support of anti-weight discrimination policies compared to adults from the other three countries, with support ranging from 28.9% to 91.5%. The current study reported support of the same policies ranging from 31.3% to 76.9%. In both studies, the same two policies received the lowest and highest level of support. However, support for some policies may have been higher in the previous study due to bias from the recruited sample who were members of Obesity Canada (formerly known as the Canadian Obesity Network), a professional organization aimed at improving the lives of Canadians living with obesity and reducing weight bias and stigma through research, advocacy and education (39). Members surveyed in the previous multinational study included “allied health professionals, policymakers, industry stakeholders, teachers, research trainees, media representatives, mental health professionals, and administrators” (21). Moreover, similar to this current study, the majority of participants supported policies that address workplace weight-discrimination.

Attributing the causes of obesity to psychological factors has previously been shown to significantly predict support of policies. Moreover, having anti-fat attitudes such as *Blame* (i.e., blaming the individual for their weight status) and *Willpower* (i.e., believing obesity is a matter of willpower and personal control) significantly predicted opposition to these policies (21). These previous results align with the findings of the current study, whereby attributing the causes of obesity to the behavioral and non-behavioral factors predicted support of 8 of these anti-weight discrimination policies. Furthermore, *Dislike* and *Willpower* were associated with a reduced likelihood of supporting six and two of these policies, respectively, a majority of which were employment-related anti-weight discrimination policies. This signifies that the more Canadian adults dislike individuals with overweight and obesity and attribute obesity to personal responsibility and lack of willpower, the less likely they are to support policies aimed at reducing weight discrimination, especially in the workplace. Attribution theory (38, 40, 41) explains that individuals make sense of behaviors by understanding the causes of such behaviors (41). Based on this, believing that weight is not necessarily within one's personal control would likely allow for a reduction in blaming the individual. Therefore, educational efforts are needed to inform the Canadian public on the non-behavioral causes of obesity to reduce blame and explicit weight bias and limit the attribution of obesity to the willpower of the individual. This could ultimately help increase the likelihood of supporting policies to reduce weight discrimination. In this study, we attempted to understand the relationship between attributing obesity to behavioral and non-behavioral causes (COB questionnaire) and the likelihood of supporting these anti-weight discrimination policies. We hypothesized, that non-behavioral causes would predict greater support, while behavioral causes would predict a lower level of support. While our results showed that attributing obesity to non-behavioral causes predicted greater policy support, unexpectedly, attributing obesity to behavioral causes also predicted support. Even though beliefs about the causes of obesity are associated with weight bias, it is possible that these beliefs alone cannot invoke negative attitudes extreme enough to oppose policies that advocate for the rights of a certain population. It may be that some of the behavioral causes included (such as physical inactivity or overeating) were not extreme enough, as compared to explicit attitudes such as dislike, to invoke opposition of policies that can protect individuals from weight discrimination. We can speculate that the questionnaire itself, which covers an array of causes of obesity, allows respondents to recognize the multifaceted and complex nature of obesity and therefore support potential policies to protect them from discrimination. We suspect that measures of beliefs about causes of obesity could be improved by incorporating more evidence-based causes of obesity that have been identified in more recent years than the current measure used. Future studies should continue to examine the relationship between beliefs about other causes of obesity and supporting policies aimed at reducing weight discrimination.

In the current study, WBI predicted support for broad societal policies but not the employment-related policies aimed at reducing weight discrimination. Given that individuals across the weight spectrum can experience WBI, regardless of experiencing weight stigma from others, it is possible that the value of societal-based policies is higher than that of employment-based policies. If individuals cannot relate to experiences of workplace weight discrimination, they may not support polices aimed at reducing these instances. Future research should measure experiences of weight discrimination in different settings and explore reasons for why people support or oppose certain anti-weight discrimination policies. Individual BMI may also play a role in why people support or oppose these policies, as having a higher BMI predicted less support of workplace weight discrimination. Future research should also examine the moderating role of BMI with these predictors. Moreover, WBI was significantly associated with a decreased likelihood of supporting policies aimed protecting individuals from workplace weight discrimination. Only one other previously conducted study utilized WBI as a predictor for support for anti-weight discrimination policies, in a sample of German adults (29). This previous study did not observe a relationship between WBI and policy support. This is likely because the previous study utilized a condensed three-item version of the Weight Bias Internalization Scale whereas our study was the first known study to utilize a more comprehensive measure of WBI by using all but one item from the WBIS-M. Weight bias becomes internalized when individuals believe the negative stereotypes surrounding obesity to be true and apply them to themselves, further influencing their perceived social adequacy or self-efficacy (42). Perhaps individuals who experience WBI may believe that weight discrimination is warranted. Internalizing weight bias may help explain why an individual chooses to oppose policies aimed at reducing workplace weight discrimination. Future research should focus on levels of WBI within the workplace and its relationship with support of workplace-related anti-weight discrimination policies.

One policy that requires additional attention is policy 4, which states that obesity should be classified as a disability to seek protection against discrimination. There are controversies associated with this policy as some individuals who live with overweight or obesity may not be considered as living with a disability according to the Americans with Disabilities Act (ADA) definition (21). Moreover, not all weight-based discrimination can be classified as discrimination due to perceived disability, therefore many individuals would not be protected against discrimination through this means (21). Despite the controversies associated with this policy, there has been public advocacy to establish obesity as a disability within North America. In 2009, the U.S. Congress passed the Americans with Disabilities Amendments (ADA) Act which included “severe obesity” as a new form of disability (43). In Canada, according to the Ontario Human Rights Commission and the Human Rights Tribunal of Ontario, in the past, obesity has not been considered a disability under the Ontario Human Rights Code unless it was caused by “bodily injury, birth defect or illness” (44). However, in 2010, “severe obesity” was included as a disability within the Code (45). In this study, only 38.5% of the sample supported this policy, which was relatively low compared to the support received for the other policies aimed at reducing weight discrimination (the average percentage of participants who supported policies 1–10 was 55.6%). The lower level of support could be attributed to the fact that some people are opposed to classifying obesity as a disability because they believe doing so might result in more discrimination and mental health issues for those classified as having a disability (46). Support for this policy has been shown to be greater among those with higher levels of obesity. In a sample of 1,000 German adults with obesity, 38.2% of participants supported establishing obesity as a disability, however those classified as having class II obesity were significantly more likely to support this policy compared to those with lower levels of obesity (47). The same trend was present in the current study, whereby those with obesity were significantly more likely to support policy 4 compared to those classified as having normal weight. More research is needed to better understand the reasons for supporting or opposing this policy and potential implications of classifying obesity as a disability on weight bias and discrimination of people living with obesity.

This research provides useful evidence to inform public health action by describing public support of policies that aim to protect individuals from discrimination based on their weight and size. Public support of anti-weight discrimination policies ranged from 31.3% to 76.9, illustrating that Canadians strongly support protecting the human rights of individuals living with large bodies. Weight bias, stigma and discrimination has already been identified as a human rights (1, 48) and social justice issue (49, 50). This study adds to our existing knowledge by demonstrating that Canadians support policies to protect people from discrimination based on their physical weight and size. This research provides important evidence for advocates, researchers, and public health practitioners to create campaigns, rallies, and gather community support with aims of introducing a bill to amend the Human Rights Codes of different jurisdictions to enact change on this social justice issue. Moreover, this research provides evidence of public support for such policies to protect against weight discrimination that should be taken forth to policy makers to enact change in Canada.

Although weight bias has been recognized as a human rights and social justice issue (1, 48–50) with well-documented mental and physical health consequences (51, 52), we have yet to see large shifts in attitudes and practices about weight and toward people with higher weights at the institutional and societal levels. Although research evidence included herein helps inform public health action, education and awareness of weight bias is not enough to alter societal norms (53–56). Despite being outside the scope of this paper, upstream systemic approaches (57) are needed to reduce weight bias and warrant future research to determine effective strategies to improve attitudes beyond the individual level.

The results of this study expand our knowledge on the level of support as well as the predictors of such support for anti-weight discrimination policies in a large sample of Canadian adults. We recognize that public support of policies does not necessarily translate into policy implementation. However, these results contribute to the evidence base on public opinion regarding protection against weight discrimination and could help inform advocacy efforts aiming to recognize weight bias, stigma, and discrimination as a violation to human rights and a social justice issue (57–59). In addition, the findings of this study may inform future research to determine the impact of implementing these publicly supported policies on the prevalence of weight-discrimination and the impact it might have on the lives of individuals with obesity. Results gathered from these studies could potentially motivate policy makers to enact policies protecting individuals with obesity from experiencing weight discrimination. Despite the strengths of the study and the novelty of the results, this study was cross-sectional by nature, thus neither causality nor directionality can be attributed to the reported relationships. Understanding these predictors at several instances through a longitudinal study design could provide important data on how attitudes might change and evolve over time. Although we did not measure sexual orientation and (dis)ability, these are important variables to consider in future research examining predictors of support for anti-weight-discrimination policies. It is incumbent for future research to explore the intersectionality of such factors like BMI, race, gender, sexual orientation and (dis)ability when it comes to support of policies that protect people from weight discrimination.

## 5. Conclusion

To conclude, this study demonstrated Canadian public support of policies aimed at reducing discrimination toward individuals with overweight and obesity, within society and the workplace. Government officials and policy makers should begin working toward including weight as a potential form of discrimination that should be addressed. Moreover, advocacy should continue to be pursued to better inform those in positions of power on the prevalence and dangers of weight discrimination, and the public's desire to create changes within society to improve the lives of those who continue to experience weight discrimination.

## Data availability statement

Data will be made available upon request. Requests to access these datasets should be directed to angela.alberga@concordia.ca.

## Ethics statement

The studies involving human participants were reviewed and approved by Concordia University Human Research Ethics Committee. The patients/participants provided their written informed consent to participate in this study.

## Author contributions

We certify that this manuscript is solely the work of ML, VF, IYE, and ASA. All authors contributed substantially to the study design, acquisition of data, data analysis, manuscript drafting, and seen and approved the final manuscript.
