# Supplementary material for: Predictors of support for anti-weight discrimination policies among Canadian adults
Source: Front Public Health. 2023 Apr 17;11:1060794. doi: 10.3389/fpubh.2023.1060794 (PMC10149811; doi:10.3389/fpubh.2023.1060794)
Supplement: Supplementary file 1 [file Table_1.docx]

**Table 3.** Predictors of Support of Anti-weight Discrimination Policies

| **Policy** | **OR [95% CI]** |
| --- | --- |
| ***Policy 1 – My country should include body weight in our civil rights law in order to protect people from discrimination based on their body weight*** |  |
| Gender |  |
| Men vs. Women | 0.71 [0.54, 0.92]* |
| Age |  |
| 25-44 vs. 18-24 | 1.29 [0.81, 2.04] |
| 45-65 vs. 18-24 | 1.24 [0.78, 1.96] |
| 65+ vs. 18-24 | 1.32 [0.77, 2.26] |
| Race |  |
| White vs. Non-White | 1.08 [0.80, 1.46] |
| Income |  |
| $25,000-49,999 vs. <$25,000 | 1.13 [0.77, 1.67] |
| $50,000-74,999 vs. <$25,000 | 0.68 [0.45, 1.02] |
| $75,000+ vs. <$25,000 | 0.76 [0.51, 1.11] |
| Body Weight |  |
| BMI ^a^ | 1.04 [1.02, 1.06]*** |
| Underweight vs. Normal Weight | 0.93 [0.51, 1.70] |
| Overweight vs. Normal Weight | 1.40 [1.01, 1.92]* |
| Obesity vs. Normal Weight | 1.50 [1.07, 2.11]* |
| Causes of Obesity ^b^ |  |
| Behavioural Causes | 1.48 [1.22, 1.78]**** |
| Non-Behavioural Causes | 1.89 [1.57, 2.28]**** |
| Anti-Fat Attitudes ^b^ |  |
| Dislike | 1.01 [0.94, 1.09] |
| Fear of Fat | 1.09 [1.04, 1.15]*** |
| Willpower | 0.97 [0.91, 1.03] |
| Weight Bias Internalization ^b^ | 1.17 [1.06, 1.29]** |
|  |  |
| ***Policy 2 – The government should have specific laws in place to protect people from weight discrimination*** |  |
| Gender |  |
| Men vs. Women | 0.83 [0.64, 1.07] |
| Age |  |
| 25-44 vs. 18-24 | 1.06 [0.68, 1.67] |
| 45-65 vs. 18-24 | 1.23 [0.79, 1.92] |
| 65+ vs. 18-24 | 1.51 [0.89, 2.57] |
| Race |  |
| White vs. Non-White | 0.89 [0.66, 1.20] |
| Income |  |
| $25,000-49,999 vs. <$25,000 | 1.02 [0.69, 1.49] |
| $50,000-74,999 vs. <$25,000 | 0.90 [0.61, 1.34] |
| $75,000+ vs. <$25,000 | 0.75 [0.51, 1.10] |
| Body Weight |  |
| BMI ^a^ | 1.03 [1.01, 1.05]** |
| Underweight vs. Normal Weight | 1.17 [0.65, 2.09] |
| Overweight vs. Normal Weight | 1.34 [0.98, 1.84] |
| Obesity vs. Normal Weight | 1.81 [1.29, 2.53]*** |
| Causes of Obesity ^b^ |  |
| Behavioural Causes | 1.42 [1.18, 1.71]*** |
| Non-Behavioural Causes | 1.94 [1.61, 2.33]**** |
| Anti-Fat Attitudes ^b^ |  |
| Dislike | 0.92 [0.86, 0.99]* |
| Fear of Fat | 1.07 [1.02, 1.13]** |
| Willpower | 0.97 [0.91, 1.03] |
| Weight Bias Internalization ^b^ | 1.14 [1.04, 1.26]** |
|  |  |
| ***Policy 3 – The government should penalize (or fine) those who discriminate against persons because of their weight*** |  |
| Gender |  |
| Men vs. Women | 0.87 [0.67, 1.13] |
| Age |  |
| 25-44 vs. 18-24 | 0.96 [0.63, 1.56] |
| 45-65 vs. 18-24 | 1.30 [0.83, 2.04] |
| 65+ vs. 18-24 | 1.76 [1.04, 3.01]* |
| Race |  |
| White vs. Non-White | 1.03 [0.77, 1.39] |
| Income |  |
| $25,000-49,999 vs. <$25,000 | 0.90 [0.61, 1.33] |
| $50,000-74,999 vs. <$25,000 | 0.78 [0.53, 1.17] |
| $75,000+ vs. <$25,000 | 0.91 [0.62, 1.34] |
| Body Weight |  |
| BMI ^a^ | 1.02 [1.00, 1.04] |
| Underweight vs. Normal Weight | 0.79 [0.44, 1.41] |
| Overweight vs. Normal Weight | 1.10 [0.80, 1.50] |
| Obesity vs. Normal Weight | 1.29 [0.92, 1.80] |
| Causes of Obesity ^b^ |  |
| Behavioural Causes | 1.23 [1.03, 1.47]* |
| Non-Behavioural Causes | 1.55 [1.30, 1.84]**** |
| Anti-Fat Attitudes ^b^ |  |
| Dislike | 0.94 [0.88, 1.01] |
| Fear of Fat | 1.03 [0.98, 1.08] |
| Willpower | 0.90 [0.85, 0.96]*** |
| Weight Bias Internalization ^b^ | 1.10 [1.00, 1.21]* |
|  |  |
| ***Policy 4 – Obesity should be considered a disability so that people will be protected from weight discrimination in the workplace*** |  |
| Gender |  |
| Men vs. Women | 1.00 [0.75, 1.32] |
| Age |  |
| 25-44 vs. 18-24 | 1.39 [0.81, 2.38] |
| 45-65 vs. 18-24 | 1.77 [1.04, 3.00]* |
| 65+ vs. 18-24 | 3.04 [1.68, 5.51]*** |
| Race |  |
| White vs. Non-White | 1.08 [0.78, 1.49] |
| Income |  |
| $25,000-49,999 vs. <$25,000 | 1.00 [0.67, 1.50] |
| $50,000-74,999 vs. <$25,000 | 0.90 [0.60, 1.37] |
| $75,000+ vs. <$25,000 | 0.61 [0.40, 0.92]* |
| Body Weight |  |
| BMI ^a^ | 1.03 [1.01, 1.05]** |
| Underweight vs. Normal Weight | 0.99 [0.51, 1.92] |
| Overweight vs. Normal Weight | 1.32 [0.94, 1.87] |
| Obesity vs. Normal Weight | 1.63 [1.14, 2.33]** |
| Causes of Obesity ^b^ |  |
| Behavioural Causes | 1.29 [1.06, 1.57]* |
| Non-Behavioural Causes | 1.82 [1.49, 2.22]**** |
| Anti-Fat Attitudes ^b^ |  |
| Dislike | 1.10 [1.02, 1.19]* |
| Fear of Fat | 1.03 [0.98, 1.09] |
| Willpower | 0.94 [0.88, 1.01] |
| Weight Bias Internalization ^b^ | 1.20 [1.08, 1.33]*** |
|  |  |
| ***Policy 5 – Fat persons should be subject to the same legal protections and benefits offered to people with physical disabilities*** |  |
| Gender |  |
| Men vs. Women | 1.15 [0.88, 1.50] |
| Age |  |
| 25-44 vs. 18-24 | 2.10 [1.23, 3.61]** |
| 45-65 vs. 18-24 | 2.65 [1.55, 4.52]*** |
| 65+ vs. 18-24 | 4.86 [2.66, 8.87]**** |
| Race |  |
| White vs. Non-White | 1.24 [0.91, 1.69] |
| Income |  |
| $25,000-49,999 vs. <$25,000 | 1.12 [0.76, 1.66] |
| $50,000-74,999 vs. <$25,000 | 1.00 [0.67, 1.50] |
| $75,000+ vs. <$25,000 | 0.85 [0.57, 1.25] |
| Body Weight |  |
| BMI ^a^ | 1.03 [1.01, 1.05]** |
| Underweight vs. Normal Weight | 0.87 [0.46, 1.65] |
| Overweight vs. Normal Weight | 1.53 [1.10, 2.13]** |
| Obesity vs. Normal Weight | 1.90 [1.34, 2.68]*** |
| Causes of Obesity ^b^ |  |
| Behavioural Causes | 1.05 [0.87, 1.27] |
| Non-Behavioural Causes | 1.44 [1.20, 1.72]**** |
| Anti-Fat Attitudes ^b^ |  |
| Dislike | 1.07 [1.00, 1.16] |
| Fear of Fat | 1.01 [0.96, 1.07] |
| Willpower | 0.96 [0.90, 1.02] |
| Weight Bias Internalization ^b^ | 1.14 [1.04, 1.26]** |
|  |  |
| ***Policy 6 - The government should pass the Weight Discrimination in Employment Act to protect employees from discrimination in the workplace based on their body weight*** |  |
| Gender |  |
| Men vs. Women | 0.83 [0.64, 1.07] |
| Age |  |
| 25-44 vs. 18-24 | 1.18 [0.75, 1.86] |
| 45-65 vs. 18-24 | 1.64 [1.04, 2.57]* |
| 65+ vs. 18-24 | 1.91 [1.12, 3.25]* |
| Race |  |
| White vs. Non-White | 1.12 [0.82, 1.50] |
| Income |  |
| $25,000-49,999 vs. <$25,000 | 1.06 [0.72, 1.57] |
| $50,000-74,999 vs. <$25,000 | 0.82 [0.55, 1.23] |
| $75,000+ vs. <$25,000 | 0.82 [0.56, 1.20] |
| Body Weight |  |
| BMI ^a^ | 1.04 [1.02, 1.06]*** |
| Underweight vs. Normal Weight | 0.70 [0.38, 1.29] |
| Overweight vs. Normal Weight | 1.43 [1.05, 1.97]* |
| Obesity vs. Normal Weight | 1.71 [1.22, 2.40]** |
| Causes of Obesity ^b^ |  |
| Behavioural Causes | 1.35 [1.13, 1.63]** |
| Non-Behavioural Causes | 1.86 [1.55, 2.24]**** |
| Anti-Fat Attitudes ^b^ |  |
| Dislike | 0.93 [0.86, 1.00]* |
| Fear of Fat | 0.99 [0.94, 1.04] |
| Willpower | 0.92 [0.86, 0.98]** |
| Weight Bias Internalization ^b^ | 1.02 [0.92, 1.12] |
|  |  |
| ***Policy 7 - It should be illegal for an employer to refuse to hire a qualified person because of his or her body weight*** |  |
| Gender |  |
| Men vs. Women | 0.56 [0.43, 0.74]**** |
| Age |  |
| 25-44 vs. 18-24 | 1.02 [0.65, 1.61] |
| 45-65 vs. 18-24 | 1.77 [1.12, 2.80]* |
| 65+ vs. 18-24 | 2.45 [1.38, 4.34]** |
| Race |  |
| White vs. Non-White | 1.44 [1.06, 1.96]* |
| Income |  |
| $25,000-49,999 vs. <$25,000 | 1.01 [0.67, 1.51] |
| $50,000-74,999 vs. <$25,000 | 0.94 [0.62, 1.42] |
| $75,000+ vs. <$25,000 | 0.97 [0.65, 1.45] |
| Body Weight |  |
| BMI ^a^ | 1.03 [1.01, 1.05]** |
| Underweight vs. Normal Weight | 0.58 [0.33, 1.05] |
| Overweight vs. Normal Weight | 1.43 [1.03, 1.99]* |
| Obesity vs. Normal Weight | 1.60 [1.12, 2.28]** |
| Causes of Obesity ^b^ |  |
| Behavioural Causes | 1.45 [1.19, 1.75]*** |
| Non-Behavioural Causes | 1.70 [1.40, 2.05]**** |
| Anti-Fat Attitudes ^b^ |  |
| Dislike | 0.86 [0.80, 0.93]**** |
| Fear of Fat | 1.04 [0.98, 1.09] |
| Willpower | 0.94 [0.88, 1.00] |
| Weight Bias Internalization ^b^ | 1.07 [0.97, 1.19] |
|  |  |
| ***Policy 8 - It should be illegal for an employer to assign lower wages to a qualified employee because of his or her body weight*** |  |
| Gender |  |
| Men vs. Women | 0.60 [0.44, 0.82]** |
| Age |  |
| 25-44 vs. 18-24 | 1.30 [0.79, 2.12] |
| 45-65 vs. 18-24 | 1.71 [1.04, 2.80]* |
| 65+ vs. 18-24 | 3.02 [1.55, 5.87]** |
| Race |  |
| White vs. Non-White | 1.85 [1.33, 2.58]*** |
| Income |  |
| $25,000-49,999 vs. <$25,000 | 1.54 [0.99, 2.39] |
| $50,000-74,999 vs. <$25,000 | 1.27 [0.81, 1.98] |
| $75,000+ vs. <$25,000 | 1.85 [1.19, 2.89]** |
| Body Weight |  |
| BMI ^a^ | 1.04 [1.01, 1.06]** |
| Underweight vs. Normal Weight | 0.57 [0.31, 1.04] |
| Overweight vs. Normal Weight | 1.41 [0.98, 2.05] |
| Obesity vs. Normal Weight | 1.83 [1.21, 2.78]** |
| Causes of Obesity ^b^ |  |
| Behavioural Causes | 1.75 [1.41, 2.17]**** |
| Non-Behavioural Causes | 1.75 [1.41, 2.16]**** |
| Anti-Fat Attitudes ^b^ |  |
| Dislike | 0.86 [0.79, 0.93]*** |
| Fear of Fat | 1.07 [1.00, 1.13]* |
| Willpower | 1.05 [0.97, 1.13] |
| Weight Bias Internalization ^b^ | 0.98 [0.87, 1.10] |
|  |  |
| ***Policy 9 - It should be illegal for an employer to terminate or fire a qualified employee because of his or her body weight*** |  |
| Gender |  |
| Men vs. Women | 0.58 [0.43, 0.78]*** |
| Age |  |
| 25-44 vs. 18-24 | 1.81 [1.14, 2.88]* |
| 45-65 vs. 18-24 | 2.64 [1.65, 4.22]**** |
| 65+ vs. 18-24 | 3.42 [1.87, 6.26]**** |
| Race |  |
| White vs. Non-White | 1.74 [1.26, 2.40]*** |
| Income |  |
| $25,000-49,999 vs. <$25,000 | 1.49 [0.98, 2.27] |
| $50,000-74,999 vs. <$25,000 | 1.64 [1.06, 2.55]* |
| $75,000+ vs. <$25,000 | 1.56 [1.03, 2.38]* |
| Body Weight |  |
| BMI ^a^ | 1.03 [1.01, 1.06]* |
| Underweight vs. Normal Weight | 0.65 [0.36, 1.18] |
| Overweight vs. Normal Weight | 1.75 [1.22, 2.52]** |
| Obesity vs. Normal Weight | 1.55 [1.06, 2.27]* |
| Causes of Obesity ^b^ |  |
| Behavioural Causes | 1.64 [1.33, 2.02]**** |
| Non-Behavioural Causes | 1.54 [1.26, 1.88]**** |
| Anti-Fat Attitudes ^b^ |  |
| Dislike | 0.86 [0.80, 0.94]*** |
| Fear of Fat | 1.05 [0.99, 1.12] |
| Willpower | 0.99 [0.93, 1.07] |
| Weight Bias Internalization ^b^ | 1.00 [0.89, 1.11] |
|  |  |
| ***Policy 10 - It should be illegal for an employer to deny a promotion or appropriate compensation to a qualified employee because of his or her body weight.*** |  |
| Gender |  |
| Men vs. Women | 0.49 [0.35, 0.66]**** |
| Age |  |
| 25-44 vs. 18-24 | 1.18 [0.73, 1.93] |
| 45-65 vs. 18-24 | 1.71 [1.04, 2.80]* |
| 65+ vs. 18-24 | 2.50 [1.32, 4.74]** |
| Race |  |
| White vs. Non-White | 1.97 [1.42, 2.73]**** |
| Income |  |
| $25,000-49,999 vs. <$25,000 | 1.46 [0.94, 2.27] |
| $50,000-74,999 vs. <$25,000 | 1.18 [0.76, 1.83] |
| $75,000+ vs. <$25,000 | 1.53 [0.99, 2.37] |
| Body Weight |  |
| BMI ^a^ | 1.02 [1.00, 1.04] |
| Underweight vs. Normal Weight | 0.54 [0.30, 0.99]* |
| Overweight vs. Normal Weight | 1.30 [0.90, 1.88] |
| Obesity vs. Normal Weight | 1.34 [0.90, 1.99] |
| Causes of Obesity ^b^ |  |
| Behavioural Causes | 1.77 [1.43, 2.19]**** |
| Non-Behavioural Causes | 1.71 [1.39, 2.10]**** |
| Anti-Fat Attitudes ^b^ |  |
| Dislike | 0.85 [0.79, 0.92]*** |
| Fear of Fat | 1.06 [0.99, 1.12] |
| Willpower | 0.99 [0.92, 1.06] |
| Weight Bias Internalization ^b^ | 0.97 [0.86, 1.08] |
|  |  |
| ***Policy 11 - Individual companies should have the right to determine whom to hire based on an employee's personal body weight*** |  |
| Gender |  |
| Men vs. Women | 1.39 [1.02, 1.90]* |
| Age |  |
| 25-44 vs. 18-24 | 1.35 [0.79, 2.32] |
| 45-65 vs. 18-24 | 0.90 [0.52, 1.55] |
| 65+ vs. 18-24 | 1.07 [0.57, 2.03] |
| Race |  |
| White vs. Non-White | 0.67 [0.48, 0.94]* |
| Income |  |
| $25,000-49,999 vs. <$25,000 | 0.90 [0.57, 1.42] |
| $50,000-74,999 vs. <$25,000 | 1.01 [0.64, 1.62] |
| $75,000+ vs. <$25,000 | 0.98 [0.62, 1.54] |
| Body Weight |  |
| BMI ^a^ | 0.98 [0.95, 1.00]* |
| Underweight vs. Normal Weight | 0.90 [0.46, 1.76] |
| Overweight vs. Normal Weight | 0.82 [0.57, 1.18] |
| Obesity vs. Normal Weight | 0.60 [0.40, 0.90]* |
| Causes of Obesity ^b^ |  |
| Behavioural Causes | 1.16 [0.93, 1.44] |
| Non-Behavioural Causes | 1.11 [0.91, 1.36] |
| Anti-Fat Attitudes ^b^ |  |
| Dislike | 1.35 [1.24, 1.47]**** |
| Fear of Fat | 1.07 [1.00, 1.13]* |
| Willpower | 1.07 [1.00, 1.15] |
| Weight Bias Internalization ^b^ | 1.18 [1.06, 1.33]** |
|  |  |
| ***Policy 12 - Employers should be allowed to assign different salaries to employees based on their body weight*** |  |
| Gender |  |
| Men vs. Women | 1.18 [0.82, 1.71] |
| Age |  |
| 25-44 vs. 18-24 | 1.54 [0.84, 2.83] |
| 45-65 vs. 18-24 | 0.60 [0.32, 1.16] |
| 65+ vs. 18-24 | 0.74 [0.34, 1.59] |
| Race |  |
| White vs. Non-White | 0.49 [0.33, 0.71]*** |
| Income |  |
| $25,000-49,999 vs. <$25,000 | 1.18 [0.69, 2.03] |
| $50,000-74,999 vs. <$25,000 | 1.30 [0.76, 2.25] |
| $75,000+ vs. <$25,000 | 0.78 [0.44, 1.38] |
| Body Weight |  |
| BMI ^a^ | 1.00 [0.98, 1.03] |
| Underweight vs. Normal Weight | 1.23 [0.56, 2.70] |
| Overweight vs. Normal Weight | 1.15 [0.74, 1.78] |
| Obesity vs. Normal Weight | 0.99 [0.61, 1.59] |
| Causes of Obesity ^b^ |  |
| Behavioural Causes | 1.09 [0.85, 1.40] |
| Non-Behavioural Causes | 1.18 [0.93, 1.50] |
| Anti-Fat Attitudes ^b^ |  |
| Dislike | 1.33 [1.21, 1.47]**** |
| Fear of Fat | 1.05 [0.98, 1.13] |
| Willpower | 1.03 [0.95, 1.12] |
| Weight Bias Internalization ^b^ | 1.38 [1.21, 1.58]**** |
| Key  *a* = adjusting for age, gender, and race  *b* = adjusting for age, gender, race, and BMI |  |
